# Mechanism of cancer stemness maintenance in human liver cancer

**DOI:** 10.1038/s41419-022-04848-z

**Published:** 2022-04-21

**Authors:** Ning Liang, Tao Yang, Qian Huang, Pengfei Yu, Chaoxu Liu, Liusheng Chen, Qian Wang, Gang Wang, Xianli He

**Affiliations:** 1 Department of General Surgery, Tangdu Hospital, Air Force Military Medical University, Xi’an, Shaanxi China; 2 Department of General Surgery, The 75th Group Army Hospital, Dali, China; 3 Department of Pain Treatment, Tangdu Hospital, Air Force Military Medical University, Xi’an, China; 4 Department of Obstetrics and Gynecology, The 75th Group Army Hospital, Dali, China; 5 State Key Laboratory of Cancer Biology and National Clinical Research Center for Digestive Diseases, Xijing Hospital of Digestive Diseases, Air Force Military Medical University, Xi’an, Shaanxi Province China; 6 Department of General Surgery, the First Affiliated Hospital of Zhejiang University, Hangzhou, China; 7 Clinical Medical Research Center, The 75th Group Army Hospital, Dali, China; 8 Department of Anorectal Surgery, The First Affiliated Hospital, Zhengzhou University, Zhengzhou, China; 9 Department of General Surgery, the 74th group army hospital, Guangzhou, China

**Keywords:** Cancer stem cells, Tumour biomarkers, Prognostic markers, Cancer stem cells

## Abstract

Primary liver cancer mainly includes the following four types: hepatocellular carcinoma (HCC), cholangiocarcinoma (CCA), hepatoblastoma (HB), and combined hepatocellular carcinoma and cholangiocarcinoma (cHCC-CCA). Recent studies have indicated that there are differences in cancer stem cell (CSC) properties among different types of liver cancer. Liver cancer stem cells (LCSCs), also called liver tumor-initiating cells, have been viewed as drivers of tumor initiation and metastasis. Many mechanisms and factors, such as mitophagy, mitochondrial dynamics, epigenetic modifications, the tumor microenvironment, and tumor plasticity, are involved in the regulation of cancer stemness in liver cancer. In this review, we analyze cancer stemness in different liver cancer types. Moreover, we further evaluate the mechanism of cancer stemness maintenance of LCSCs and discuss promising treatments for eradicating LCSCs.

## Facts

- There are significant differences in the stemness of different types of liver cancer (i.e., HCC, CCA, HB, and cHCC-CCA).
- Enhanced mitophagy and mitochondrial fission promote stemness maintenance in liver cancer.
- The crosstalk between LCSCs and tumor microenvironment (TME) promotes stemness maintenance in HCC.
- The inflammatory cytokine microenvironment promotes retrodifferentiation and stemness maintenance in liver cancer.
- Inhibitors targeting m^6^A modification enzymes will be a promising therapy for the eradication of LCSCs.

## Open questions

- How do LCSCs and their niche interact?
- Are m^6^A-related proteins, such as WTAP, FTO, and YTHDF1 (i.e., m^6^A writers, erasers, and readers), involved in the regulation of liver cancer stemness?
- What are the special conditions or incentives for the transformation of normal liver stem cells into LCSCs? Do these conditions include environmental factors, genetic factors, or both?
- What is the effect and mechanism of tumor immunotherapy on LCSCs?

## Introduction

Liver cancer stem cells (LCSCs) are a small subset of tumor cells with high self-renewal ability, strong tumor initiation potential, and unlimited differentiation ability [1]. It is believed that LCSCs are resistant to conventional radiotherapy or chemotherapy. Moreover, LCSCs have been proposed to be a driving force in tumor recurrence and metastasis [2]. However, the mechanism of LCSC stemness maintenance is very complex and has not been fully clarified thus far. On the one hand, increasing evidence shows that the stemness maintenance of LCSCs depends on a supportive tumor microenvironment, indicating that dynamic “crosstalk” occurs between LCSCs and various components in the tumor microenvironment [3–5]. On the other hand, the differentiation plasticity of tumor cells can also promote the stemness maintenance of LCSCs [6]. Under the changed tumor microenvironment and therapeutic pressure, non-CSCs can retrodifferentiate or transdifferentiate into LCSCs to supplement the CSC pool [6].

In this review, we systematically summarize the mechanisms of cancer stemness maintenance of LCSCs, including tumor plasticity, epigenetic regulation, metabolic rewiring and tumor microenvironment. We also discuss various therapeutic approaches for eradicating LCSCs, which may be the most effective and promising therapy for liver cancer in the future.

## LCSC markers and key pathways and noncoding RNAs involved in stemness maintenance

Research over the past several decades has demonstrated some markers used to identify CSC populations. Several well-established tumor stem cell markers and potential novel markers are summarized in Supplementary Table S1. Supplementary Table S2 summarizes the role of some recently identified noncoding RNAs in liver cancer stemness maintenance. Some well-known signaling pathways involved in the maintenance of stemness in liver cancer are listed in Supplementary Table S3.

## Cancer stemness in different types of liver cancer

Primary liver cancer mainly includes the following four types: hepatoblastoma (HB), hepatocellular carcinoma (HCC), cholangiocarcinoma (CCA) and combined hepatocellular carcinoma and cholangiocarcinoma (cHCC-CCA) [7].

**Fig. 1:**
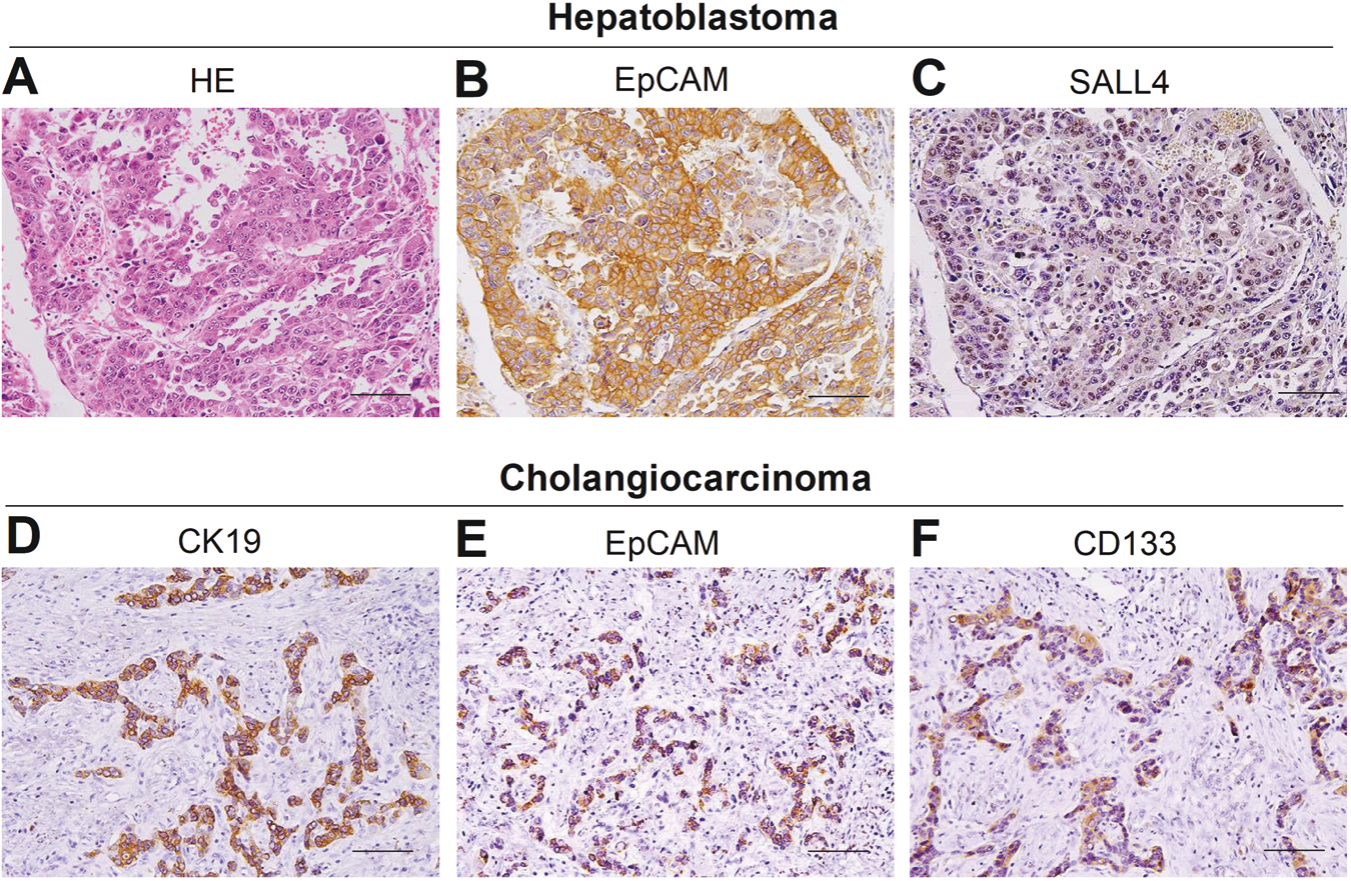
HB and CCA are two kinds of liver cancer with remarkable stemness features. **A** HE and **B**, **C** IHC showed that stemness markers (EpCAM and SALL4) were positive in HB. **D**–**F** IHC showed that stemness markers (CK19, EpCAM, and CD133) were positive in CCA. *HE* hematoxylin-eosin staining, *IHC* immunohistochemical staining.

**Fig. 2:**
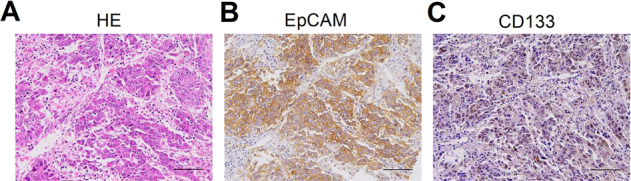
*c-MYC*-induced HCC has strong cancer stemness properties. *c-MYC*-induced HCC sections were subjected to **A** HE and **B**, **C** immunohistochemical staining. *HE* hematoxylin-eosin.

HB, which originates from hepatic primordial embryonic cells, accounts for over 80% of malignant liver tumors in children, and its incidence has been increasing in recent years. Although curative surgery combined with chemotherapy has improved the prognosis of children with HB, the prognosis of advanced-stage HB is remains very poor, and the 3-year disease-free survival rate is <34% [8]. Our previous studies have shown that stemness markers (EpCAM and SALL4) are strongly expressed in HB, indicating it as a type of liver cancer with remarkable stemness properties (Fig. 1A–C and Suppl. Fig. 1). In agreement with this, *c-MYC*-driven HB-like liver tumors are characterized by high stemness features (Fig. 2). Consistently, a recent study revealed that aberrant expression of CSC markers (CD44, CD90, and CD133) is significantly associated with disease progression and reduced survival in patients with HB [9]. Our analyses may also indicate several potential markers for HB based on the GSE131329 and GSE132037 datasets (Suppl. Fig. 1), which should be identified in the future. Recent studies by Marayati et al. [10] have indicated that inhibition of PIM kinases decreases HB cancer cell stemness and sensitizes drug-resistant HB cells to cisplatin chemotherapy. In addition, Wnt pathway activation is crucial for CSC proliferation and self-renewal in HB [11], indicating that Wnt pathway inhibitor administration can serve as an effective strategy to treat HB.

Interestingly, HCC show more significant heterogeneity, including CSCs [12], which might also be suggested from the results of our analyses based on TCGA-LIHC, GSE76247, and GSE76279 (Suppl. Fig. 2). Specifically, our analytical results showed that the mRNA levels of the identified LCSC markers varied significantly in the tumor group, including those markers exhibiting significant expression differences between the paratumor and tumor groups. Of course, the genes expression is regulated not only at the transcriptional level but also at the post-transcription, translation, and post-translation levels. Thus, our analyses indicate that we need to consider the multi-dimensional expression regulation process when exploring LCSCs. Moreover, our previous study showed that the difference in brain-expressed X-linked protein 1 (*BEX1*) expression could exhibit different stemness features and pathological phenotypes of HB or HCC [13]. Similarly, *DVL1* expression and SIRT1/MRPS5 axis activity might govern the LCSC phenotype of cell biological function [14, 15]. Importantly, a recent study based on the technology of single cell transcriptomics has shown that different subpopulations of LCSCs exist in tumors [12], which indicates the necessity to further explore the classification of different subsets of LCSCs and their corresponding biological functions. The rapid progress of single-cell multiomics technology will help us expound the comprehensions of more dimensions in the process of HCC malignant tumorigenesis and metastasis and their effects on tumorigenesis and development alone or interactively.

CCA is a highly invasive tumor that is characterized by the presence of a large proportion of CSCs. Moreover, many stemness markers, such as biliary markers (CK19), EpCAM, and SALL4, are highly expressed in CCA (Fig. 1D–F and Suppl. Fig. 3). Lorenzo et al. recently identified DCLK1 as a novel stem cell marker in human CCA, representing a serum biomarker for early CCA diagnosis [16]. Several studies have revealed a complicated mechanism of CCA stemness maintenance. Huang et al. demonstrated that inhibitor of differentiation 3 (ID3) promotes stem cell features by increasing the transcriptional activity of β-catenin [17]. Interestingly, cancer-associated fibroblasts (CAFs) enhance the stemness of CCA by recruiting myeloid-derived suppressor cells (MDSCs) via 5-lipoxygenase [18]. Furthermore, a specific subset of stem-like macrophages has been reported to help CSCs promote CCA progression, suggesting a rationale for a synergistic therapeutic strategy for CCA disease [19]. However, the mechanism underlying stemness maintenance in CCA has not been sufficiently clarified.

**Fig. 3:**
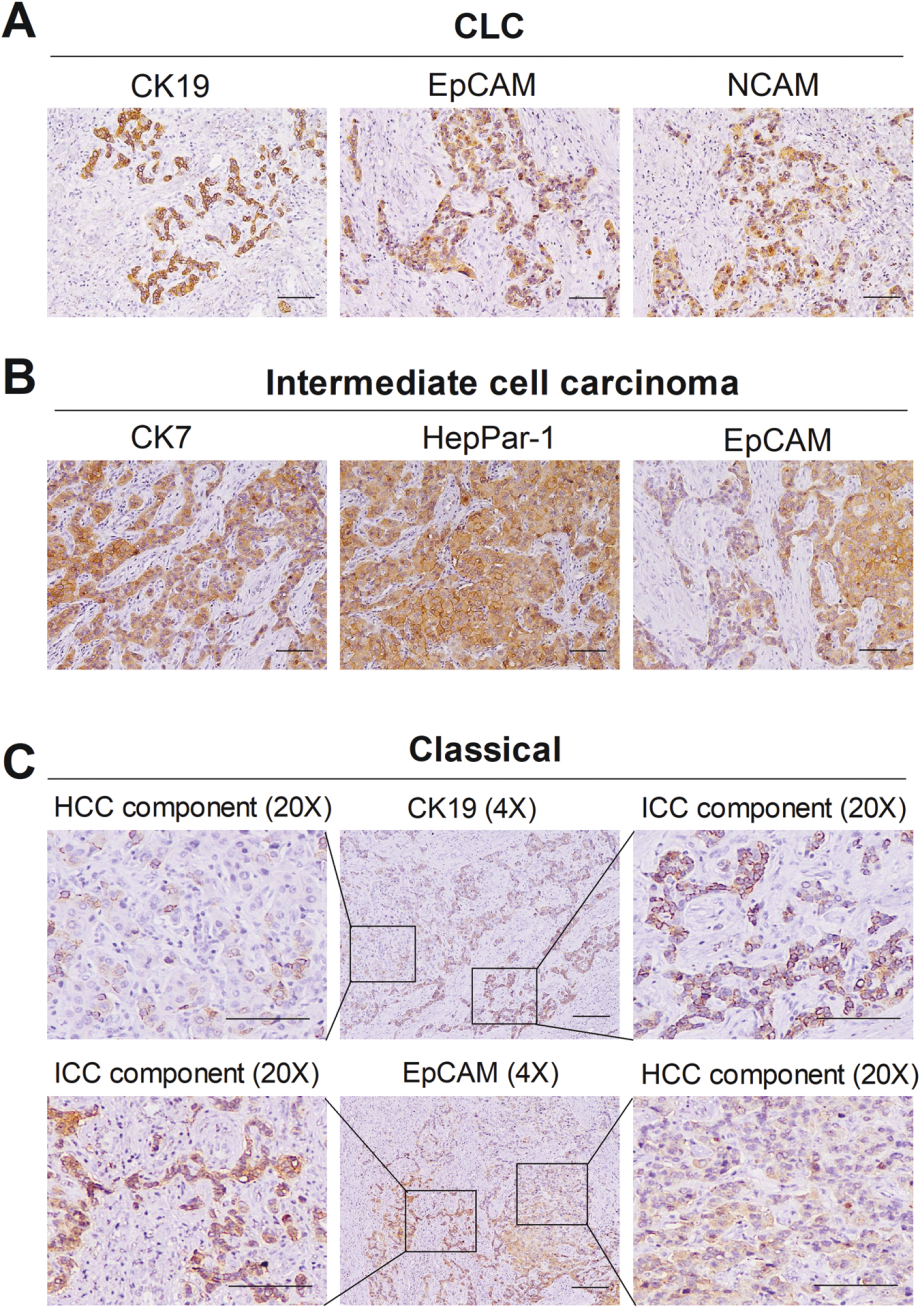
Immunostaining profile of cHCC-CCA subclasses. Representative positive immunohistochemical staining features observed for **A** the CLC type, **B** intermediate cell carcinoma and **C** the classic type. *CLC* cholangiolocellular carcinoma.

cHCC-CCA is a rare primary liver malignancy that accounts for 1–14.2% cases [20]. The diagnosis of cHCC-CCA is based on histologic features of both hepatocellular and biliary epithelial elements that are intimately admixed. It is almost impossible to make an accurate preoperative diagnosis of cHCC-CCAs using tumor markers or abdominal imaging methods. Owing to the lack of an established consensus method for pathological diagnosis, the probability of misdiagnosis is greatly increased. According to the World Health Organization (WHO) classification of 2019, cHCC-CCA is mainly divided into two types: classic and intermediate cell carcinomas [20]. The classic type shares a biphenotypic fingerprint and has both HCC and CCA components, with an intermediate transition that is characterized by two mixed entities. Intermediate cell carcinoma is now defined by the presence of homogeneous biphenotypic tumor cells, whereas the condition previously termed cholangiolocellular carcinoma (CLC) does not belong to cHCC-CCA and represents a separate biliary-derived entity with no genomic features of HCC [20]. We found that CLC tumors were positive for CK19 and progenitor-like markers (EpCAM and NCAM) (Fig. 3A), which is in agreement with the findings of a previous study [20]. Moreover, CLC is associated with chromosomal stability and transforming growth factor-β (TGF-β) pathway activation. In addition, the CXCL12-CXCR4 axis, which has been described to be closely associated with stemness in many tumors [21], has also been shown to be significantly upregulated in CLC.

Intermediate-cell carcinomas were positive for both the cholangiocytic marker CK7 and the hepatocytic marker HepPar-1. Moreover, the expression of EpCAM, a stemness marker, was strongly positive in intermediate cell carcinomas (Fig. 3B). In addition, intermediate-cell carcinomas are associated with poor prognosis and the activation of stemness-related pathways, such as the MYC, IGF, mTORC, OCT4, and NOTCH signaling pathways. Regarding the classic type, the CCA component has stronger CSC properties than the HCC component (Fig. 3C). Likewise, a recent study has also revealed that the classic CCA component has a more aggressive phenotype than the HCC component. Furthermore, integrative genomic analysis of the classic CCA component indicated the activation of proliferative signals, proinflammatory pathways and promitotic DNA replication-related signaling [19].

## Plasticity of cancer stemness

Plasticity is defined as phenotypic transformation, the dedifferentiation of differentiated cells into stem/progenitor cells, or conversion of various differentiated cell states. We discuss herein some examples of stem cell plasticity associated with tumor initiation and progression, such as epithelial–mesenchymal transition (EMT) and differentiation plasticity.

### Epithelial–mesenchymal transition

EMT is a complicated process that involves cellular and molecular reprogramming. In this process, epithelial cells lose their differentiated features and acquire mesenchymal properties, including invasiveness and motility. Recently, EMT was found to serve as a vital regulator of cancer cells exhibiting CSC-like features. For example, Mitra et al. revealed that cell surface Vimentin (csVim), an intracellular EMT tumor cell marker, expression could be used to isolate cancer stem-like cells [22]. They found that csVim(+)CD133(−) cells had stem-like features similar to those of the csVim(−)CD133(+) population [22]. Similarly, Li et al. documented that CD90(+) LCSCs have high Vimentin expression [23]. Another study demonstrated that CD44(+) LCSCs express low levels of the epithelial marker E-cadherin and high levels of the mesenchymal markers (i.e., N-cadherin and Vimentin) [24]. Moreover, CD44 downregulation reverses the EMT process by inhibiting the ERK/Snail pathway, thereby preventing lung metastasis in a mouse metastasis model [24]. The close link between EMT and LCSCs may form the basis for finding new targeted drugs to provide novel treatments for tumors. One possible method is to target LCSC surface antigens that control EMT. Recent studies have demonstrated that downregulation of CD133 or CD44 eliminates HCC cells with CSC and EMT phenotypes. Another approach is to screen compounds that can cause cell death of LCSCs generated by EMT.

**Fig. 4:**
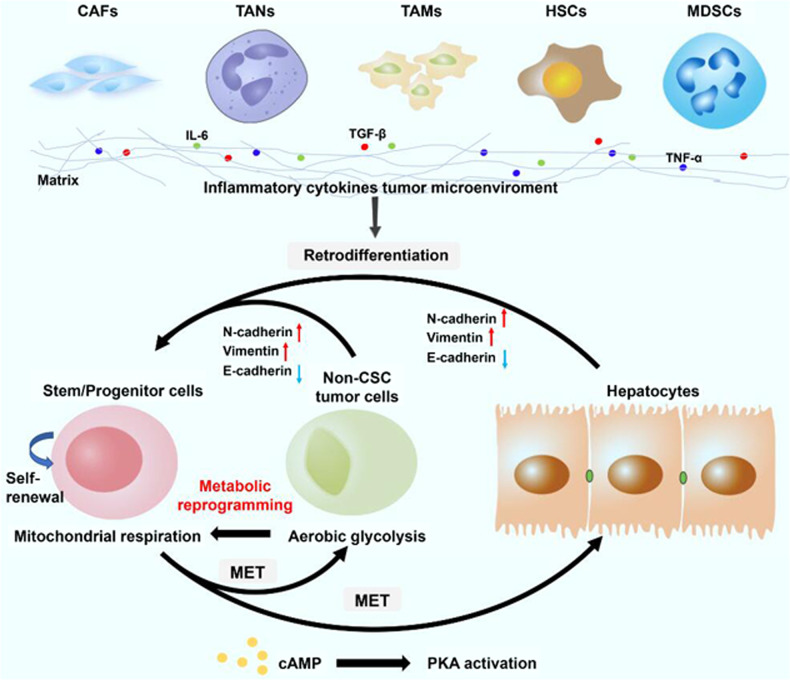
The inflammatory cytokine tumor microenvironment promotes retrodifferentiation and cancer stemness maintenance in liver cancers. The process of retrodifferentiation includes EMT and metabolic reprogramming of non-CSC cells. Additionally, activation of PKA can induce MET in CSCs and lead to the loss of tumor-initiating ability in CSCs. *CSCs* cancer stem cells, *EMT* epithelial–mesenchymal transition, *MET* mesenchymal-to-epithelial transition, *CAFs* cancer-associated fibroblasts, *TANs* tumor-associated neutrophils, *TAMs* tumor-associated macrophages, *HSCs* hepatic stellate cells, *MDSCs* myeloid-derived suppressor cells, *PKA* protein kinase A.

In recent years, promoting mesenchymal–epithelial transition (MET), the reverse process of EMT, has been proposed as a strategy to eradicate mesenchymal tumor-initiating cells (TICs) by inducing their transformation into epithelial cells that lack stemness properties. For instance, the activation of protein kinase A (PKA) can induce MET in TICs in a histone demethylase PHF2-dependent manner, leading to the loss of tumor-initiating ability in TICs [25]. Another study demonstrated that the Chinese herbal compound “Songyou Yin” restored the epithelial marker E-cadherin and reduced the expression of mesenchymal marker Vimentin [26]. These results indicate that MET induction could serve as a differentiation therapy for LCSCs in the future (Fig. 4).

### Differentiation plasticity

Cell plasticity, including retrodifferentiation and transdifferentiation of differentiated cells, facilitates supplementation of the CSC pool with non-CSCs. Accumulating evidence suggests that an inflammatory environment promotes the retrodifferentiation of tumor cells into stem/progenitor cells, which may be partly responsible for chemotherapy resistance and tumor recurrence [27]. Notably, a recent study has shown that NF-κB, a key inflammatory regulator, is involved in the retrodifferentiation of tumor-derived hepatocytes into hepatic progenitor cells [6]. Other cytokines, such as TNF-α, IL-6, CCL22, and TGF-β, can also promote the retrodifferentiation of tumor-derived hepatocytes into stem/progenitor cells [6]. Moreover, NF-κB pathway inhibition by the IKK inhibitor curcumin restrains stem-like features and sensitizes liver cancer cells to the HDAC inhibitor trichostatin. IL-6 can also contribute to the retrodifferentiation of HCC cells into LCSCs through the JAK1-STAT3-OCT4 pathway. IL-6 also increases the sphere formation ability and the expression of stemness-related genes in many human HCC cell lines. Other cytokines, such as TGF-β, TNF-α, IL-1β, IL-8, and IL-11 can also promote the retrodifferentiation of tumor-derived hepatocytes into stem/progenitor cells [6]. Once retrodifferentiated, these cells continue to produce the aforementioned cytokines to maintain the CSC-like phenotype. In conclusion, an inflammatory environment can trigger or enhance the retro-differentiation of non-CSCs into LCSCs to promote recurrence after conventional chemotherapy. Thus, targeting inflammatory regulators and relevant signaling pathways can effectively inhibit the acquisition and maintenance of the LCSC-like phenotype. However, given the close association between inflammation and protective immunity, these strategies might increase the risk of infection. Therefore, attenuation, rather than inhibition, of the inflammatory response should be considered.

In our previous study, we showed that HCC, intrahepatic CCA (ICC), and cHCC-ICC could originate from mature hepatocytes in three mouse liver cancer models using hydrodynamic transfection methodology [7]. Thus, we speculate that mature hepatocytes transdifferentiate into hepatic progenitor cells in a specific TME and then differentiate into various types of liver cancer. This speculation is supported by a recent study published in *Nature* [27]. In that study, the authors demonstrated that a necroptosis-associated hepatic cytokine microenvironment determines the transdifferentiation of mature hepatocytes into ICC, whereas an apoptotic microenvironment promotes the transdifferentiation of mature hepatocytes into HCC [27] (Fig. 4). In view of the emerging role of differentiation plasticity in the maintenance of the CSC-like phenotype, inducing the differentiation of CSCs into non-CSCs to attenuate their stemness features using differentiating agents will be an effective treatment. Yamashita et al. reported that oncostatin M, an interleukin 6-related cytokine, can induce the hepatocytic differentiation of EpCAM(+) LCSCs [28]. Similarly, Yin et al. demonstrated that overexpression of HNF4α promoted the differentiation of HCC cells into hepatocytes and significantly reduced the percentage of CD133 (+) LCSCs [29]. Zhang et al. showed that high-dose exogenous BMP4 promotes CD133 + LCSC differentiation and inhibits their tumorigenic capacity [30]. Future studies determining novel and effective differentiation agents are urgently needed to eradicate LCSCs.

## Epigenetic regulation and liver cancer stemness

### DNA methylation

The importance of epigenetics in liver cancer has been gradually recognized. In the process of HBV-related multistep hepatocarcinogenesis, methylation events mainly occur in the early stage of hepatocarcinogenesis [31]. LCSCs play vital roles in cancer initiation, therapy resistance, recurrence, and metastasis. Recently, accumulating evidence has suggested that DNA methylation is an important epigenetic regulatory mechanism that affects the CSC properties of liver cancer [32]. Methylation is mainly mediated by three DNA methyltransferases, namely, DNMT3a, DNMT3b and DNMT1 [33]. Raggi et al. reported that zebularine (ZEB), a DNMT1 inhibitor, causes a significant increase in the self-renewal potential of HCC cells [32]. Similarly, Mikkelsen et al. demonstrated that compared with the control treatment, inhibition of DNMT1 by 5-aza-cytidine (AZA) can lead to an increased number of embryonic stem cell-like colonies, thereby facilitating the reprogramming process [34]. The possible reason is that inhibition of DNA methylation can induce the expression of many stem cells and pluripotency-related genes by generating an active chromatin structure.

Our own work suggests that BEX1, a novel CSC marker, can be reactivated by using the DNMT1 inhibitor zebularine, further indicating the importance of epigenetic mechanisms for LCSCs [13]. Therefore, in the future, an in-depth understanding of the epigenomic mechanisms in CSCs will help us to develop effective anti-CSC therapies.

### N-methyladenosine (m^6^A) RNA methylation

**Fig. 5:**
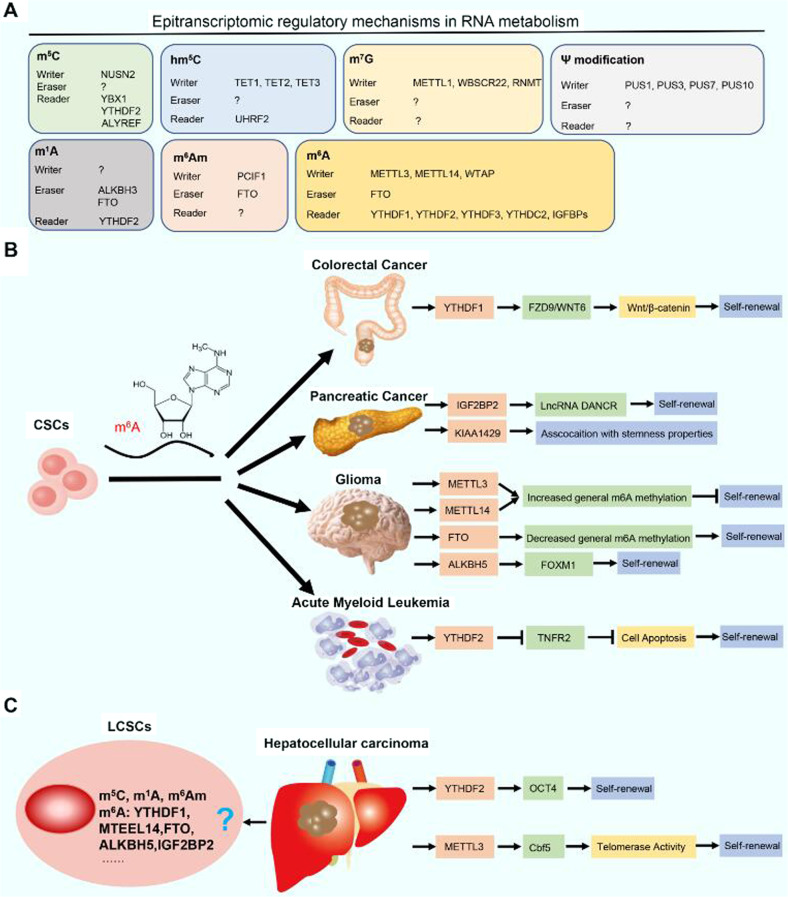
Roles of m^6^A modifications in LCSCs. **A** Epitranscriptomic regulatory mechanisms in RNA metabolism. The roles of m^6^A-related enzymes in (**B**) different CSCs and (**C**) LCSCs are shown. Abbreviations: CSCs, cancer stem cells; LCSCs, liver cancer stem cells.

To date, at least 100 kinds of chemical RNA modifications, such as N6-methyladenosine (m^6^A), 5-methylcytosine (m^5^C), N6,2′-O-dimethyladenosine (m^6^Am), and 5-hydroxymethylcytidine (hm^5^C), have been identified, and together, these modifications constitute the epitranscriptome [35]. The biological effects of m^6^A modification have been well demonstrated; nevertheless, the biological functions of other RNA modifications, such as m^5^C, m^6^Am, and hm^5^C, remain unclear [35]. Herein, we focused on the role of m^6^A in CSCs, especially in LCSCs. The role of N6 methyladenosine (m^6^A) RNA methylation in regulating the LCSC phenotype has attracted increasing attention. For instance, YTHDF1 has been found to be overexpressed in colonospheres and to regulate the self-renewal of CSCs through the Wnt/β-catenin pathway in colorectal cancer [36]. Zhang et al. demonstrated that the m^6^A demethylase ALKBH5 promotes the proliferation and tumorigenesis of CSCs by maintaining FOXM1 expression in glioblastoma [37]. In pancreatic cancer, insulin-like growth factor 2 mRNA-binding protein 2 (IGF2BP2), which represents a unique family of m6A readers, promotes cell proliferation and stemness maintenance of CSCs [38]. Most notably, YTHDF2, an m^6^A reader, has been shown to promote the stemness phenotype in HCC [39]. On the one hand, knockout of YTHDF2 in LCSCs reduces the m^6^A level in the 5’-untranslated region of OCT4 mRNA and then reduces the expression of the OCT4 protein, a pivotal transcription factor modulating the stemness and malignant development of liver cancer [39] On the other hand, YTHDF2 promotes m^6^A-mediated mRNA degradation of suppressor of cytokine signaling 2 (SOCS2, a cancer-suppressive factor). Another study demonstrated that CircMEG3 inhibits malignant differentiation and telomerase activity in LCSCs by reducing Cbf5 (a component of telomere synthetase) through a m^6^A methyltransferase METTL3 dependent mechanism [40]. In the future, more studies are required to further elucidate whether other proteins, such as YTHDF1, ALKBH5, IGF2BP2, and FTO (i.e., m6A writers, erasers and readers), are involved in the regulation of liver cancer stemness. Based on the m^6^A machinery, we believe that more effective therapeutic approaches can be developed to completely eradicate both cancer cells and LCSCs (Fig. 5).

## Metabolic rewiring

Many studies have revealed that LCSCs depend on rewired metabolic processes to maintain their stemness properties. Metabolism rewiring helps LCSCs adapt to the TME and facilitate tumor progression. The metabolic phenotype of LCSCs mainly enhances mitochondrial oxidative phosphorylation, which differs from the main role of aerobic glycolysis in differentiated non-CSCs [15]. Interestingly, a specific glycolysis phenotype of *MYC*-induced LCSCs exists [41], which indicates that different conditions could drive diverse metabolic phenotypes. Moreover, relative to glucose, fatty acid oxidation produces more energy at the same molar ratio. It has also been reported that LCSCs can maintain stemness by enhancing fatty acid oxidation while inhibiting mitochondrial oxidative phosphorylation activity in the condition of *NANOG*-mediated stemness maintenance and nonalcoholic fatty liver disease (NAFLD)-HCC initiation [42, 43]. In addition to powering LCSCs, unsaturated fatty acids can also promote tumor cell membrane fluidity [44], which contributes to tumor progression and metastasis. Moreover, it has been validated that unsaturated fatty acids produced by stearoyl-CoA desaturase1 (SCD1), the key rate-limiting enzyme for lipid desaturation, could enhance stemness phenotype of tumor initiating stem cell-like cells by amplifying Wnt-β-catenin signaling. Therefore, the reduction in unsaturated fatty acid synthesis by inhibiting SCD1 can inhibit the stemness of CSCs and improve the sensitivity of CSCs to other antitumor drugs in many tumors, such as LCSCs [45]. In addition, it is generally believed that there is a close relationship between unsaturated fatty acids in the cell membrane and ferroptosis [46], an iron-dependent, lipid-peroxide-driven form of programmed cell death that differs, from other forms of cell death. Of note, lethal lipid peroxide (LPO) is the main cause of ferroptosis; hence, eliminating LPO can protect cells from ferroptosis. The elimination of LPO depends on the NADPH/FSP1/coenzyme Q10 (CoQ10) and system X_C_^−^/GSH/GPX4 axes [46]. In fact, inhibiting the expression of some key regulators in the LPO process, such as system X_C_^−^, FSP1 and GPX4, has been proven to effectively inhibit the CSC stemness phenotype by inducing ferroptosis [47]. In particular, it needs to be pointed out that ferroptosis is a complex process, and its biological function remains unknown. Factors regulating the balance between fatty acid desaturation and ferroptosis also need to be explored. Regulating ferroptosis in LCSCs has the potential to improve HCC treatment; however, further research is needed.

Besides glucose and fatty acid metabolic rewiring, LCSCs also reprogram the processes involved in phospholipid and cholesterol metabolism. Vinciguerra et al. successively demonstrated the silencing of macroH2A1, a histone H2A variant, inducing a stem cell-like phenotype of HCC cells characterized by a lipidomic signature with increased levels of total sphingomyelin and decreased levels of total lysophosphatidylcholine [48]; moreover, single-cell RNA-sequencing and metabolomic analysis has shown that the phospholipid synthesis pathway is significantly enriched in HCC. The role of lysophosphatidylcholine acyltransferase, a key enzyme in phospholipid metabolism, should be further investigated in LCSCs [49]. In addition, it has been revealed that high cholesterol induces a notable TIC phenotype [50], indicating that targeting cholesterol biosynthesis can be used to decrease the proportion of LCSC population. Furthermore, the metabolic reprogramming of bile acids biotransformed from cholesterol contributes to the induction of LCSCs. For instance, glycochenodeoxycholic acid enhances stemness and chemoresistance via the STAT3 signaling pathway in HCC cells [51]. In NAFLD-HCC human species and mouse models, the upregulation of steroidogenic acute regulatory protein 1 can stimulate the expression of stemness-related genes by increasing bile acid profiles comprising unconjugated primary bile acids, β-muricholic acid, cholic acid, and their tauroconjugates [52]. Thus, an obvious question that needs to be answered is how phospholipids, cholesterol, and other metabolites participate in the modulation of LCSC stemness maintenance under different HCC-inducing conditions.

**Fig. 6:**
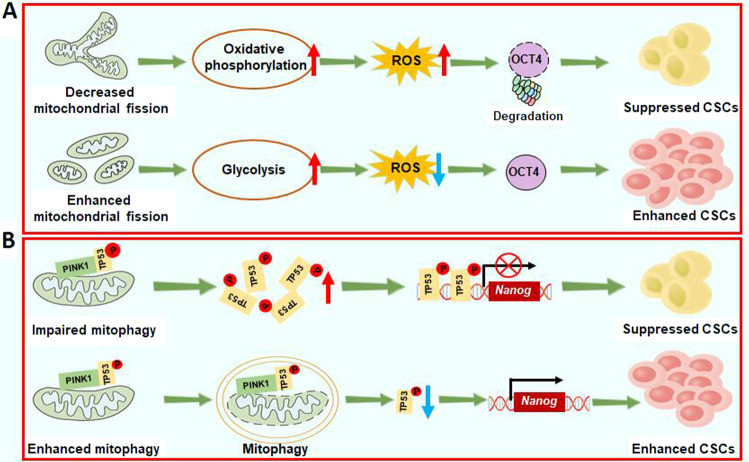
Enhanced mitochondrial fission and mitophagy promote cancer stemness maintenance in liver cancer. **A** Enhanced mitochondrial fission promotes metabolic switching from oxidative phosphorylation to glycolysis, thereby reducing ROS-mediated degradation of OCT4 and promoting cancer stemness maintenance in liver cancer. **B** When mitophagy is enhanced, p53 phosphorylated by PINK1 is degraded by mitophagy, leading to increased expression of NANOG. In contrast, impaired mitophagy promotes the nuclear localization of TP53 and then inhibits the self-renewal ability of LCSCs.

Mitochondrial dynamics, which are regulated by constant cycles of mitochondrial fusion and fission, contribute to metabolic rewiring and tumor progression in many types of cancers. Recent research has indicate that mitochondrial fission caused by mitochondrial fission factor can induce mitophagy and asymmetric stem cell division and subsequently regulate metabolic switching from oxidative phosphorylation to glycolysis, thereby reducing the production of mitochondrial reactive oxygen species (ROS) and suppressing ROS-mediated degradation of the pluripotency marker OCT4 [53] (Fig. 6A**)**. These results demonstrate that mitochondrial fission-mediated metabolism rewiring promotes the maintenance of stemness in HCC; thus, the inhibition of mitochondrial fission-mediated metabolism rewiring may be an attractive therapeutic option for HCC.

Thus, metabolic flexibility could be the Achilles heel of LCSC [1]. For instance, the restoration of oxidative phosphorylation activity and inhibition of fatty acid oxidation hindered the self-renewal of LCSCs and reduced drug resistance [42]. Similarly, metformin, which affects cellular energy metabolism, can dramatically decrease the number of EpCAM (+) LCSCs via the AMPK/mTOR pathway [54]. In addition, the inhibition of ATP-binding cassette (ABC) transporters, such as ABCB1/MDR1 and ABCG2/BCRP, represents a promising strategy for eliminating LCSCs [55]. Another approach is to perform high-throughput screening to identify valuable targets that inhibit the energy metabolism of LCSCs. Some inhibitors of oxidative phosphorylation, such as natural compound analogs (leucinostatin, oligomycin, antimycin, and efrapeptin), have been shown to effectively suppress the self-renewal of liver SALL4(+) LCSCs [56].

It should also be noted that LCSCs undergo metabolic switching dynamics according to extrinsic signals. Measuring the process and promoting its clinical application is an oncoming problem. The study by Rafael et al. provids a novel single-cell resolution energy metabolism function analysis method based on flow cytometry named SCENITH, which can divide cell populations into four metabolic subtypes, profoundly characterize different metabolic states, and interpret LCSC heterogeneity [57]. In addition, rapidly progressing microscopic technology shows tremendous potential for revealing the complexity of metabolic rewiring, such as the metabolic activity phenotype method using multiple vibrational metabolic probes [58] and two-photon microscopy combined with specific near-infrared fluorescent probes reflecting metabolic patterns [59].

## The TME and liver cancer stemness maintenance

**Fig. 7:**
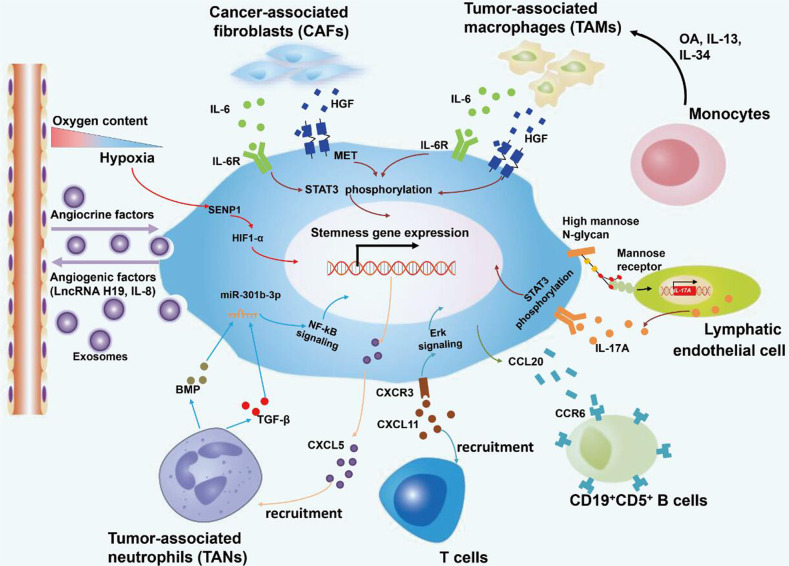
The crosstalk between LCSCs and cellular components in the TME promotes stemness maintenance in HCC. Cellular components in the TME include tumor-associated macrophages, monocytes, fibroblasts, neutrophils, hepatic stellate cells, LECs, T cells, and B cells. Moreover, angiogenesis and the hypoxic microenvironment promote stem cell gene expression and tumorigenesis. *OA* osteoactivin.

The TME in liver cancer includes the extracellular matrix (ECM) and cellular components. In this section, we will elaborate on the relationship between liver cancer stemness and TME (Fig. 7). Although many components of the TME contribute to the maintenance of stemness in LCSCs, there may be a complex interplay among these components. What is the most important component of TME? Will combined intervention with multiple components destroy the stemness maintenance of LCSCs? These questions need to be answered in future studies.

### ECM within TME and maintenance of CSC-like phenotype

The ECM is a complicated noncellular three-dimensional network mainly composed of glycosaminoglycan, fibronectin, collagen, proteoglycan, elastin, and other glycoproteins. It is considered to provide a niche for LCSCs. Although the mechanism is unclear, soft matrix stiffness contributes to tumor outgrowth through disseminated tumor cells. For example, THBS2-deficient CD133(+) LCSCs can form a local soft ECM by regulating collagen degradation and matrix metalloproteinase activity. The local “soft spot” microenvironment can increase the stemness and drug resistance of LCSCs and provide an escape route for metastasis [60]. Recent research using microfluidic technology has found that intrinsic softness mediated by BCL9L is a unique marker of highly tumorigenic and metastatic tumor cells. Similarly, high ECM stiffness has been reported to promote stemness maintenance in HCC by activating integrin β1/Akt/mTOR/SOX2 signaling [61]. Additionally, highly activated tumor-associated fibroblasts increase ECM stiffness and favor angiogenesis, thereby providing adequate nutrition for CSCs [62]. Hence, we speculated that ECM stiffness may depend on the different stages of tumor development. In the early stage of HCC, higher ECM stiffness enhances LCSC proliferation and self-renewal, whereas in the late stage of HCC, a softer ECM may facilitate LCSC metastasis. A significant problem is how LCSCs perceive and conduct stress from the tumor matrix. Recent progress in microfluidic devices and atomic force microscopy has shown strong application potential [63, 64]. Further investigation based on such biomechanical technologies might reveal the effects of tumor matrix stiffness on stemness maintenance, LCSC morphology, and metabolic phenotype.

#### Hypoxia within TME

It is well known that hypoxia plays an important role in the production and maintenance of stemness in a variety of tumors, including HCC. For example, liver hypoxia caused by surgery leads to the rapid dedifferentiation of tumor cells into immature CSCs with high metastatic potential [65]. Moreover, the expression of stemness- and hypoxia-related markers has recently been found to be closely related to HCC [66]. Experimental results have shown that hypoxia increases specific small ubiquitin-like modifier protease 1-mediated hypoxia-inducible factor-1α (HIF-1α) deSUMOylation to enhance HIF-1α stability and transcriptional activity [67]. Another study confirmed that ubiquitin-specific protease 22 enhances HIF-1α stability through a deubiquitination mechanism [68]. Thus, increases in HIF-1α levels contribute to the restoration of oxygen homeostasis and further promote the maintenance of stemness in LCSCs [67]. In addition, the result of a new study highlighting the significance of oxygen concentration in preclinical studies, demonstrate that primary tumors collected, treated and cultured in a hypoxic environment of 5% oxygen concentration exhibit increase expression of Lgr5, Wnt, YAP1, and EpCAM and are resistant to targeted therapy [69]. Interestingly, tumors are sensitive to several drugs previously missed under normoxic conditions of 21% oxygen concentration, indicating that further investigation on different oxygen conditions should be implemented to explore specific differences in LCSCs cultured in ambient and physioxic environments.

#### Iron within TME

Mounting evidence supports a positive correlation between increased iron storage and the risk of liver cancer, especially in patients with hemochromatosis. Generally, considering the “iron addiction” of tumor cells to maintain cell proliferation, the change in iron transport in cancer cells leads to the acquisition of iron. In the past few years, several studies have found an increase in iron content in the CSCs of several types of tumors, and also showed that altered iron metabolism is a functional requirement of CSCs in tumor growth. A recent study demonstrated that CD44 mediates the endocytosis of iron-bound hyaluronates in cancer cells and that enhanced endocytosis is accompanied by the EMT process and CSC phenotype, which indicates an intense link between CSCs and iron metabolism [70]. Meanwhile, it is worth noting that there is limited knowledge about the role of iron in LCSCs.

In addition, the most obvious signatures of iron metabolism-related ROS dynamics and complicated metabolic signaling should not be ignored. A recent study reported novel CD133(−) and EpCAM(−) targeted liposomes with redox-responsive properties capable of synergistically eliminating LCSCs. Taken together, artesunate was shown to induce ER-derived ROS-mediated cell death by disrupting the labile iron pool and iron redistribution in HCC cells. These results suggest that therapeutics targeting ROS induced by the redox-active iron pool may have a positive potential to eradicate LCSCs. A key problem that needs to be addressed is how metabolic reprogramming of LCSCs is coupled with cancer cell-specific ferroptotic responses.

Considering the role of iron in CSCs, Sacco et al. provided a model for classifying tumors into hyperinflamed and cold types according to iron content in tumors; their results indicate that hyperinflamed tumors are enriched in immune cell infiltration and are characterized by a high iron content, whereas cold tumors are poor in immune cells and are distinguished by iron deficiency [71]. Moreover, a recent bioinformatic analysis partially supports this rationale, which suggests that HCC patients with a higher infiltration abundance of immature dendritic cells, cytolytic activity and inflammation promotion have a significant tumor mutation burden and CSC characteristics [72]. Importantly, an obvious defect in the proferroptotic strategy is poor targeting with serious bone marrow injury and anemia [73]. Therefore, the “double edge sword” role of iron in antitumor function should be carefully considered. A potential assumption is whether treatments could synergistically enhance the therapeutic effects based on a precise classification considering the characteristics of the tumor and whole body, such as immune cell infiltration level and tumor stiffness.

#### Vascular niche within TME

Some studies have demonstrated a strong interaction between LCSCs and endothelial cells (ECs). CD13 is a marker of both ECs and LCSCs. By targeting CD13, both LCSCs and angiogenic ECs can be cleared, suggesting that there is a close relationship between LCSCs and ECs [74]. In addition, a clinical study has shown that higher levels of circulating endothelial progenitor cells and LCSCs suggest a worse prognosis in HCC, revealing an intense link between these components [75].

Angiogenesis produces a vascular niche secreting various instructive angiocrine factors (i.e., HGF and Wnt2) to nourish LCSCs [76]. Under physiological conditions, liver injury stimulates residual hepatic sinusoidal ECs to secrete angiocrine factors to establish a vascular niche, which is vital for nurturing normal hepatic stem/progenitor cells [77]. However, the extension of this theorem to HCC is conditional because of the significant difference in the microenvironment between normal liver cells and HCC cells. In addition, ECs regulate the stemness of CSCs. According to related studies on solid tumor CSCs, ECs not only regulate the CSC phenotype of colorectal cancer cells by secreting Jagged-1 to promote Notch signaling, but also regulate the CSC phenotype and chemotherapy resistance of colorectal cancer through AKT-mediated NANOGP8 induction, suggesting that ECs often regulate the stemness of CSCs in a paracrine manner [78, 79]. Furthermore, in vitro experiments confirmed that the use of conditioned medium from ECs can promote the phenotype of LCSCs [80]. However, several questions remain to be explored. How do transdifferentiated ECs survive under the complicated conditions of TME? What is the driving force regulating the transdifferentiation of LCSCs into vascular ECs? Is this process affected by cytokines from other factors?

In contrast, LCSCs preferentially release exosomes or angiogenic factors to activate ECs [76]. Moreover, Conigliaro et al. demonstrated that CD90(+) LCSCs, but not parental liver cancer cells, can release exosomes containing the lncRNA H19 to facilitate angiogenesis in HCC [81]. Tang et al. found that CD133(+) CSCs with enhanced IL-8 (a well-known angiogenic factor) secretion exhibit enhanced ability to induce angiogenesis and tumor initiation [82]. Subsequent studies have revealed that the neurotensin/IL-8/CXCL1/MAPK signaling cascade in CD133(+) LCSCs is critical for conferring angiogenic and tumorigenic properties to these cells [82]. Vasculogenic mimicry (VM) is a widely discovered phenomenon of tumor angiogenesis in which invasive tumor cells mimic ECs by forming blood vessels in a manner significantly different from general angiogenesis. Increasing evidence suggests that CSCs contribute to VM formation to seed themselves. An interesting reflection is whether VM also exists in LCSCs. Increased ROS levels activate the Akt/IKK signaling pathway to promote the transdifferentiation of LCSCs into ECs [83]. It has been suggested that CSCs have differentiation potential under a specific TME [84]; however, this needs to be further verified by in vivo research.

Thus, the combination of antiangiogenic therapy and targeted LCSCs in the treatment of liver cancer may be effective and promising. Several studies have suggested the possibility of this treatment, and the combined sorafenib and EMT targeting can effectively target the CSC tumor subpopulation [85]. However, more effort is necessary to identify the specific mechanism. Additionally, recent excellent work- based spatial transcriptomics technology has revealed intense contact between LCSCs and vascular infiltration, which also seems to suggest a robust relationship between the two components [86]. Together, more evidence from several novel technologies, such as spatial and single-cell transcriptomics, might provide more awareness and inspiration about the topic.

### Cellular components within TME and maintenance of CSCs-like phenotype

#### Tumor-associated macrophages (TAMs) within TME

Crosstalk between TAMs and CSCs has been found in a variety of cancers, including glioblastoma, pancreatic ductal adenocarcinoma and so on [87]. Recently, Wan et al. revealed that TAMs can be used as a potential therapeutic target for LCSCs [88]. They found that HCC TAMs promote CD44( + ) LCSC expansion and accelerate sphere production. Moreover, TAMs were injected intraperitoneally into NSG mice to establish HCC peritoneal tumors. The results demonstrated that HCC tumor cells following TAM injection exhibited a higher proportion of CD44( + ) LCSCs [88]. More importantly, Wang et al. found that interleukin 6 (IL-6) secreted by HCC TAMs promotes CSC expansion in human HCC by activating STAT3 signaling [88]. Blockade of the IL-6/STAT3 pathway using tocilizumab, an anti-IL-6 receptor antibody approved by the Food and Drug Administration, significantly inhibited TAM-mediated expansion of hepatic CD44( + ) cells [88]. In addition, S100A9, an inflammatory microenvironment-related secreted protein, is secreted by TAMs and is associated with the self-renewal of CSCs [89]. Of note, a recent study indicated that some key molecules released by cholangiocarcinoma (CCA) stem-like cells, such as IL-13, osteoactivin (OA) and IL-34, regulate the differentiation of recruited monocytes toward TAMs, which is characterized by acquisition of strong invasion and adhesion capability [19]. This suggests that the combination of in vivo administration of IL13, OA and IL34 antibodies with chemotherapy may effectively eradicate CCA-infiltrating macrophages and serve as a potential treatment for CCA [19].

#### Tumor-associated neutrophils within TME

Tumor-associated neutrophils (TANs) in the cancer microenvironment play an important role in the initiation and development of liver cancer. A recent study highlighted that TANs can promote the phenotypic switching of HCC cells into tumor cells with a strong stem-like phenotype [3]. The authors found that TAN treatment had little influence on the proliferation of CD44( + ) EpCAM(+) HCC stem cells in vitro. For non-stem cells, however, TAN treatment led to increased expression of N-cadherin and Vimentin and decreased expression of E-cadherin [3]. These results demonstrate that the enhanced stem cell properties of hepatoma cells caused by TANs may be due to an epithelial-to-mesenchymal transition of the non-stem cell population [3]. In addition, TAN-secreted BMP2 and TGF-β2, which belong to the TGF-β superfamily [90], are responsible for TAN-induced miR-301b-3p expression in HCC cells with a stem-like phenotype. The increased miR-301b-3p expression subsequently inhibits the gene expression of limbic system-associated membrane protein (LSAMP) and CYLD lysine 63 deubiquitinase (CYLD), which can lead to hyperactivation of the NF-κB pathway, increased C-X-C motif chemokine 5 (CXCL5) secretion and increased TAN infiltration [3, 91]. Therefore, the direct interaction between TANs and cancer stem-like cells controls the progression of HCC, indicating that TANs may be a target for the elimination of HCC cells with a stem-like phenotype.

#### Cancer-associated fibroblasts within TME

Cancer-associated fibroblasts (CAFs) are the main component of the tumor stroma in HCC. Increasing evidence has shown that CAFs are responsible for orchestrating the self-renewal of LCSCs through secretion of various cytokines, chemokines, and growth factors. For example, Li et al. documented that hepatocyte growth factor (HGF) and IL-6 secreted by CAFs promote the stem cell-like properties of CD24(+) cells by activating STAT3 signaling [92]. Lau et al. found that CAFs play a critical role in the modulation of LCSCs via HGF-induced activation of the c-Met/FRA1/HEY1 cascade [93]. Liu et al. confirmed that CAFs activate the Notch3 pathway and then maintain LSD1 stability by inducing LSD1 deacetylation. The high expression of LSD1 in LCSCs subsequently promotes stemness maintenance of LCSCs [94].

#### Hepatic stellate cells within TME

The communication between hepatic stellate cells and LCSCs has not yet been fully elucidated. Researchers have confirmed that liver injury can activate stellate cells to release paracrine factors and thus to promote progenitor cell expansion, which may lead to either hepatic regeneration and/or hepatocarcinogenesis [95]. Interestingly, some other studies have shown that hepatic stellate cells can transdifferentiate into progenitor cells directly, which is based on several points. First, hepatic stellate cells have remarkable phenotypic plasticity [96]; Second, the hedgehog and Wnt signaling pathways, two pathways related to stem cell differentiation, are excessively activated in stellate cells [97]; Third, hepatic stellate cells express stem cell markers (i.e., CD133) and contribute to the stem cell niche according to histological studies [98]. In conclusion, stellate cells may have the potential to transdifferentiate into liver progenitor cells; however, this is still speculated and needs further verification in the future.

#### Lymphatic endothelial cells (LECs) within TME

LECs, which together represent of the most important components in the tumor microenvironment, promote tumor cell proliferation by secreting lymphoangiocrine factors. A previous study has reported that LECs preferentially interact with CD133(+) LCSCs through direct interaction between high mannose N-glycans and mannose receptors [4]. This interaction upregulates IL-17A signaling in LECs and then helps LCSCs self-renew and escape immune attack [4]. Of note, neutralization of the IL-17A pathway inhibits the self-renewal of LCSCs [4]. Therefore, IL-17A may be a promising therapeutic target for liver cancer.

#### Immune cells within TME

C-X-C motif chemokine ligand 11 (CXCL11), a member of the chemokine superfamily, can selectively recruit activated T cells to inflammatory sites [99]. Moreover, upregulated CXCL11 promotes sphere formation and tumorigenicity in α2δ1(+) LCSCs via CXCL11/CXCR3/ERK1/2 signaling, indicating that CXCL11 may act as a communication medium between activated T cells and α2δ1(+) LCSCs [5]. A very recent study reported that CCL20 could recruit CCR6-expressing CD19(+)CD5(+) B cells. CCL20, also known as the selective chemokine ligand for CCR6, is one of the CC chemokines highly expressed in TP53-mutated HCC with an obvious stemness trait. The interaction of CCL20 and CCR6 gives rise to bidirectional communication between B lymphocytes and LCSCs [100].

## Clinical implications

Elimination of CSCs by forced differentiation may be a powerful therapeutic approach. Experimental evidence also suggests that Smad inhibitors can induce the differentiation of LCSCs, resulting in significant chemosensitization [101]. Another study has indicated that forced overexpression of hepatocyte nuclear factor-4α (HNF4α) induces the differentiation of hepatic CSCs into mature hepatocytes and significantly reduces stem cell-related gene expression and the proportion of CD90( + ) and CD133( + ) LCSCs [29]. However, how to specifically differentiate tumor stem cells rather than nontargeted cells is still a problem to be explored in the future.

An increasing number of studies have shown that immunotherapy may also be an effective strategy to target LCSCs. Indeed, it has been demonstrated that LCSCs may escape immune surveillance through a PD1/PD-L1-dependent mechanism. For example, Nishida et al. demonstrated that PD-L1( + ) HCC cells are frequently positive for stemness markers such as cytokeratin 19 (CK19) and Sal-like protein 4 (SALL4) [102]. Another study revealed that the expression of SOX2, a stemness-related transcription factor, was positively correlated with PD-L1 in HCC [103]. Further analysis confirmed that SOX2 binds directly to the PD-L1 promoter and promotes its transcription [103]. Additionally, Rong et al. demonstrated that cytokine-induced killer cell (CIK, a natural killer cell subset)-mediated immunotherapy can effectively eradicate LCSCs via NKG2δ ligand recognition [104].

Autophagy is a catabolic process that removes damaged organelles and protein aggregates for recycling and plays a critical role in maintaining cellular homeostasis [105]. Mitophagy, which selectively removes mitochondria through autophagy, plays an important role in scavenging damaged mitochondria and controlling mitochondrial quality [106]. One study directed by Magalhães-Novais and her colleagues clarified that there are increased mitophagy levels in P19 stem cells, suggesting that mitophagy contributes to the maintenance of pluripotency [107]. Notably, Liu et al. proposed that mitophagy can positively regulate hepatic CSCs by inhibiting the tumor suppressor p53 [108]. PTEN-induced putative kinase 1 (PINK1) is associated with mitophagy and phosphorylates p53 at S392. When mitophagy is enhanced, p53 phosphorylated by PINK1 is removed by mitophagy, resulting in the increased expression of NANOG. In contrast, when the function of mitophagy is impaired, p53 phosphorylated by PINK1 cannot be removed by mitophagy and is transported to the nucleus, where it binds to the NANOG promoter and inhibits the expression of NANOG, a key transcription factor that promotes the self-renewal ability of LCSCs, leading to a reduction in tumor stemness [108]. Collectively, these findings suggest that mitophagy plays an important role in maintaining the hepatic CSC population by controlling p53 activities (Fig. 6B). Thus developing novel mitophagy inhibitors will be helpful for eradicating LCSCs and inhibiting the development of liver cancer.

**Fig. 8:**
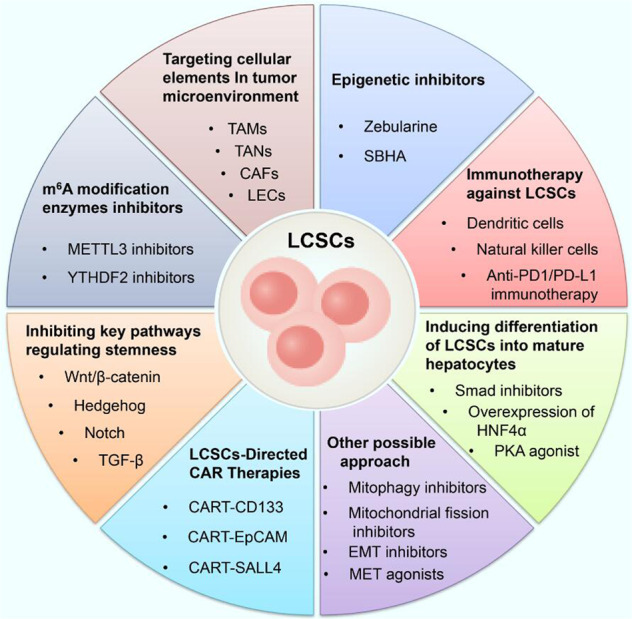
Various therapeutic approaches that may eradicate LCSCs. *LCSCs* liver cancer stem cells, *EMT* epithelial–mesenchymal transition, *MET* mesenchymal-to-epithelial transition, *TAMs* tumor-associated macrophages, *TANs* tumor-associated neutrophils, *CAFs* cancer-associated fibroblasts, *LECs* lymphatic endothelial cells, *SBHA* suberoyl bis-hydroxamic acid, *CAR-T* chimeric antigen receptor T-cell immunotherapy.

It has been demonstrated that direct targeting of hepatic CSC-specific surface markers (i.e., EpCAM, CD133, CD44 and CD90) may be an effective treatment for elimination of LCSCs. Recently, a single-arm open-label phase II trial demonstrated that CD133-targeted chimeric antigen receptor T-cell (CAR-T) cell therapy is efficacious and safe for patients with advanced HCC [109]. Immunohistochemical staining showed that CD133( + ) cells in biopsy tissue were cleared after CART-133 infusions [109]. As EpCAM is a Wnt/β-catenin signaling target [110], EpCAM-CAR-T cell therapy may potentially be useful for advanced HCC, but this possibility requires further preclinical and translational evaluation. Therefore, comprehensive therapeutic approaches eradicating large numbers of tumor cells and specific CSCs may be the most effective and promising therapies (Fig. 8).

## Conclusions

Taken together, insights into molecular alterations, tumor plasticity, epigenetic changes, and tumor environmental risk factors will shed light on potential new strategies for eliminating LCSCs. Recently, some small molecule inhibitors targeting key pathways regulating the stemness properties of LCSCs, such as the Wnt/β-catenin (NCT02069145), Hedgehog (NCT03734926), and TGF-b (NCT02906397 and NCT02240433) pathways, have begun to be tested in multiple clinical trials. Epigenetic regulation plays an important role in the initiation and progression of HCC. Some epigenetic inhibitors, such as SBHA and zebularine, can significantly affect the biological characteristics of LCSCs. However, epigenetic regulation is not limited to a specific gene and may bring about many nonspecific changes. In addition, a recent study published in *Nature* pointed out that STM2457, a small molecule inhibitor of METTL3, can significantly inhibit the progression of myeloid leukemia. Given that some m^6^A modification enzymes, such as YTHDF2 and METTL3, regulate the self-renewal of LCSCs, the development of small molecule inhibitors targeting m^6^A modification enzymes will be a promising therapy for the eradication of LCSCs. Notably, there is increasing evidence that m^6^A regulators such as ALKBH5, YTHDF2, METTL3/14 and FTO can orchestrate PD-L1 expression, indicating that m^6^A inhibitors may regulate immune responses to anti-PD-1 therapy in HCC. In recent years, with the in-depth study of LCSCs, the tumor microenvironment, mitophagy, noncoding RNAs and epithelial mesenchymal transformation have been found to be closely related to liver cancer stemness maintenance. Since LCSCs have characteristics similar to those of normal stem cells, the above mechanisms may exist in both cell subpopulations; thus, eliminating LCSCs may become a challenge. In the future, we need to further explore the different mechanisms between LCSCs and normal stem cells, which will provide insights for the development of effective therapeutics against liver cancer.

## Supplementary information

Supplemental Material

Language Editing Certificate

## Data Availability

Not applicable.
