# Supplementary material for: Mechanism of cancer stemness maintenance in human liver cancer
Source: Cell Death Dis. 2022 Apr 21;13(4):394. doi: 10.1038/s41419-022-04848-z (PMC9023565; doi:10.1038/s41419-022-04848-z)
Supplement: Supplementary file 1 — Supplemental Material [file 41419_2022_4848_MOESM1_ESM.docx]

# Supplemental information

**Supplementary Figure 1.** **The expression of stemness markers in HB.** Analyses of stemness markers expression in two HB datasets (GSE131329 and GSE132037).

**Supplementary Figure 2. The expression of stemness markers in HCC.** Analyses of stemness markers expression in TCGA-LIHC and two HCC datasets (GSE76297 and GSE76247).

**Supplementary Figure 3. The expression of stemness markers in** **CCA.** Analyses of stemness markers expression in TCGA-CHOL and two CCA datasets (GSE32879 and GSE76297).

**Suppl. Figure 1**


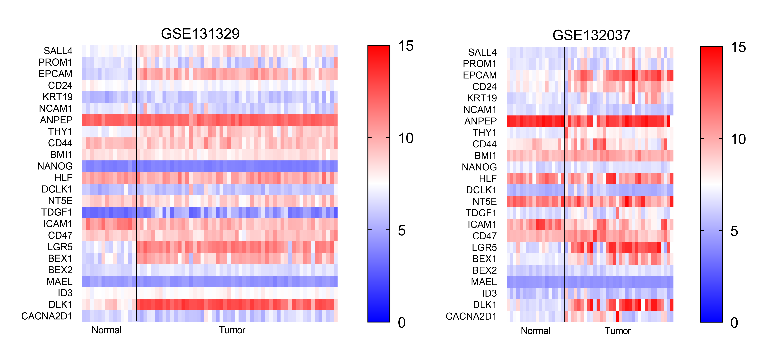


**Suppl. Figure 2**


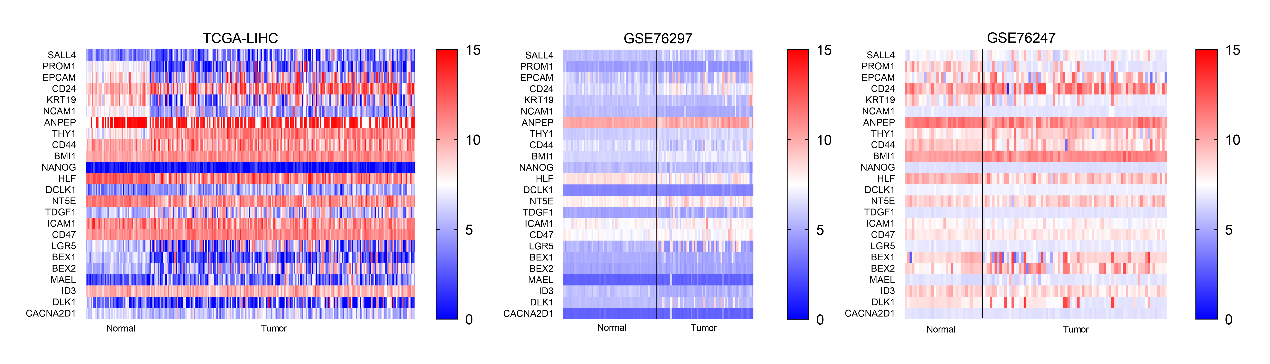


**Suppl. Figure 3**


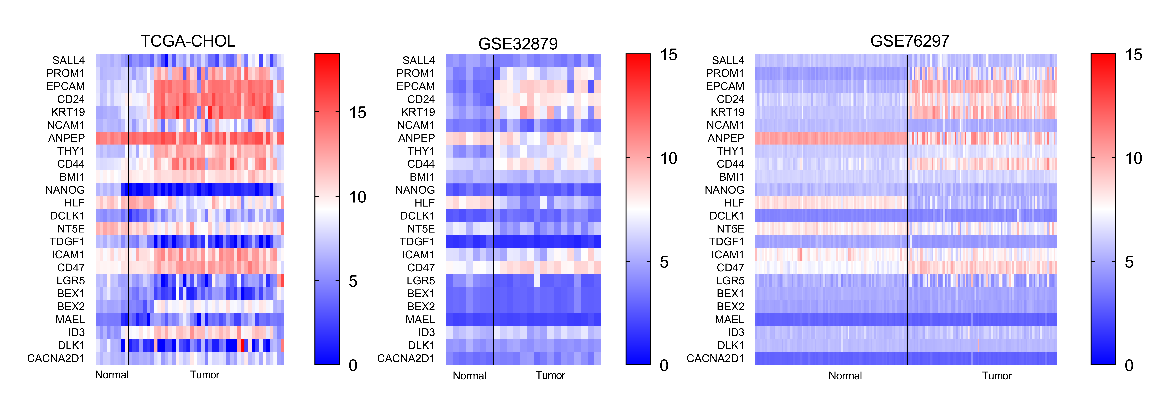


**Supplementary Tables**

**Supplementary Table S1.** **Summary of LCSC markers that have been validated by tumorigenicity assays.**

| **Well-established markers** | **Functions in LCSCs** | **Pathways involving LCSCs** | **Subcellular localisation** | **References** |
| --- | --- | --- | --- | --- |
| SALL4 | Promotes sphere formation, cell proliferation, stemness gene expression and tumorigenicity | Regulates pluripotency through interaction with OCT3/4, SOX2, and NANOG | Cell nucleus | ^1^ |
| CD133 | Promotes doxorubicin and 5-FU resistance, sphere formation and tumorigenicity | Activation of Nanog/IGF2/YAP signaling | Cell surface | ^2^ |
| EpCAM | Promotes sphere formation, tumorigenicity, invasion, 5-FU resistance, tumorigenicity | Activation of Wnt/β-catenin pathway | Cell surface | ^3^ |
| CD24 | Promotes proliferation, invasion, metastasis, and sorafenib resistance | Activation of Notch1/TACE/ADAM17 pathway | Cell surface | ^4^ |
| OV-6 | Promotes sphere formation, invasion, chemoresistance, tumorigenicity | Activation of CXCL12/CXCR4/β-catenin signaling | Cell surface | ^5^ |
| NCAM | Promotes stemness gene expression, sphere formation, tumorigenicity, invasion | Activation of Wnt/β-catenin pathway | Cell surface | ^6^ |
| CD13 | Promotes sphere formation, chemoresistance, stemness gene expression and tumorigenicity | Activation of ERK1/2  signaling pathway | Cell surface | ^7, 8^ |
| CD90 | Chemoresistance, metastasis, differentiation, and self-renewal | Activation of mTOR signaling pathway | Cell surface | ^9, 10^ |
| CD44 | Promotes stemness gene expression, sphere formation, tumorigenicity | Activation of AKT/GSK-3/-catenin, and ERK/Snail pathways | Cell surface | ^11, 12^ |
| BMI1 | Promotes sphere formation, invasion, chemoresistance, tumorigenicity | Bmi1 regulates stemness by acting as a failsafe against the p16Ink4a and p19Arf-dependent senescence pathway. | Cell surface | ^13^ |
| NANOG | Promotes sphere formation, chemoresistance, and tumorigenicity | Activation of IGF1R-signaling pathway. | Cell cytoplasm | ^14^ |
| **Potential new markers** | | | | |
| HLF | Promotes sphere formation, invasion, chemoresistance, tumorigenicity | HLF directly transactivates c-Jun pathway | Cell nucleus | ^15^ |
| DCLK1 | Promotes sphere formation, chemoresistance, and tumorigenicity | Activation of Wnt/β-catenin pathway | Cell surface | ^16^ |
| CD73 | Promotes self-renewal, differentiation, lenvatinib resistance and tumor proliferation | Upregulates the expression of SOX9 and enhancing its protein stability | Cell surface | ^17^ |
| Cripto-1 | Promotes the proliferation, migration, self-renewal ability and drug resistance | Cripto-1 stabilizes DVL3 and activates Wnt/β-catenin pathway | Cell cytoplasm | ^18^ |
| ICAM-1 | Promotes sphere formation, lung metastases, tumorigenicity | Nanog regulated expression of ICAM-1 in HCC stem cells. | Cell surface | ^19^ |
| CD47 | Promotes self-renewal, tumor initiating,  and chemoresistance | Activation of IL-6/STAT3 signaling pathway, and NF-kB | Cell surface | ^20^ |
| LGR5 | Promotes stemness gene expression, sphere formation, tumorigenicity, and sorafenib and  cisplatin resistance | Activation of Wnt/β-catenin pathway | Cell surface | ^21^ |
| BEX1 | Promotes stemness and tumorigenicity | Activation of Wnt/β-catenin signaling | Cell cytoplasm | ^22^ |
| BEX2 | Promotes sphere formation and tumorigenicity | BEX2 regulates stemness through inhibiting mitochondrial activity | Cell cytoplasm | ^23^ |
| MAEL | Promotes stemness gene expression, EMT, migration and invasion, cisplatin resistance and tumorigenicity | MAEL enhanced AKT activity with subsequent GSK-3β phosphorylation and Snail stabilization | Cell cytoplasm | ^24^ |
| ID3 | Promotes sphere formation, chemoresistance, stemness gene expression and tumorigenicity | ID3 blocks E47 recruitment to the promoter of β-catenin, leading to activation of Wnt/β-catenin signaling | Cell nucleus | ^25^ |
| DLK1 | Promotes colony formation, spheroid colony formation, chemoresistance, and tumorigenicity | Upregulates the expression of IFI16 and decreased p21waf1/cip1. | Cell surface | ^26, 27^ |
| Calcium channel α2δ1 subunit | Promotes colony formation, spheroid colony formation, and tumorigenicity | α2δ1 promotes stemness by regulating calcium influx. | Cell surface | ^28^ |

**Supplementary Table S2. Summary of MicroRNA and LncRNA and CircRNA in liver cancer stemness maintenance.**

| **Noncoding RNA** | **Function** | **Mechanisms** | **References** |
| --- | --- | --- | --- |
| **MicroRNA** |  |  |  |
| miR-125b | Induces the expression of CD24 | MiR-125b loss activated the HIF1α/pAKT loop | ^29^ |
| miR-192-5p | Suppress CSC-related features | Through the regulation of PABPC4 | ^30^ |
| miR-93 | Promotes the self-renewal of LCSCs and tumor propagation | Bind to 3'-UTR of myotubularin-related protein 3 | ^31^ |
| miR-148a | Suppress CSC-related features | Regulate ACVR1/BMP circuit | ^32^ |
| miR-1246 | Promotes the self-renewal of LCSCs and tumor propagation | Activation of the Wnt/β-catenin pathway | ^33^ |
| **LncRNA** |  |  |  |
| LncHDAC2 | Promotes the self-renewal of LCSCs and tumor propagation | Activation of Hedgehog signaling | ^34^ |
| Lnc-DILC | Promotes expansion of LCSCs | Inhibits the autocrine IL-6/STAT3 signaling | ^35^ |
| LncTCF7 | Promotes LCSC self-renewal and tumor propagation. | Recruits SWI/SNF complex to the promoter of TCF7 and activates Wnt pathway | ^36^ |
| LncHOXA10 | Promotes the self-renewal of LCSCs and tumor propagation | Recruites NURF complex to HOXA10 promoter and activates the transcription of HOXA10 | ^37^ |
| LncBRM | Promotes LCSC self-renewal and tumor propagation. | Activation of YAP1 signalling | ^38^ |
| **CircRNA** |  |  |  |
| CircZKSCAN1 | Suppresses liver cancer stemness | Inhibit the transcriptional activity of the Wnt pathway | ^39^ |
| CircRNA CDR1as | Enhances the self-renewal ability of LCSCs | Act as a miR-7-5p sponge to upregulate the expression of KLF4 | ^40^ |
| Circ-CDYL-centric | Promotes tumor propagation | Interacts with HDGF and HIF1AN by acting as the sponge of miR-892a and miR-328-3p | ^41^ |

**Supplementary Table S3. Summary of signaling pathways in liver cancer stemness maintenance.**

| **Signaling pathways** | **Function** | **Mechanisms** | **References** |
| --- | --- | --- | --- |
| c-MYC signaling | Inducing embryoid hepatoblastoma-like HCC cells and promoting stemness | Excessive mitochondrion-endoplasmic reticulum coupling;  Driving the reprogramming of micro-RNA signature | ^42^  ^43^ |
| Notch signaling | Promoting AKT/YAP and RAS-induced lineage conversion from hepatocytes to ICC formation;  Being involved in iNOS-mediated stemness maintenance of CD24(+)CD133(+) LCSCs | Notch2 signaling as well as SOX9 expression and canonical Notch targets;  Notch1/TACE/ADAM17 axis | ^44^  ^45^ |
| WNT/β-catenin signalling | Maintaining the function of hepatic CSCs;  Driving chemoresistance | Promoting the transcription of downstream target genes, such as cyclin D1, survivin and c-myc and forming a continuously enhanced positive feedback loop;  EPHB2/β-catenin/TCF1 positive feedback loop | ^46^  ^47^ |
| Hedgehog signaling | Enhancing the self-renewal and pluripotency of LCSCs | LncHDAC2 mediated PTCH1 downregulation;  Promoting hypoxic TME and potential immune privilege | ^34^  ^48^ |
| TGF-β signaling | stemness maintenance in liver cancer; | Regulating IL-6/STAT3 cascade;  Activating extracellular signal-regulated kinase (ERK) 1/2 signaling of CAFs to sustain stemness | ^49^  ^50^ |

**Supplementary References**

1. Oikawa T, Kamiya A, Zeniya M, Chikada H, Hyuck AD, Yamazaki Y*, et al.* Sal-like protein 4 (SALL4), a stem cell biomarker in liver cancers. *Hepatology (Baltimore, Md)* 2013, **57**(4)**:** 1469-1483.

2. Kohga K, Tatsumi T, Takehara T, Tsunematsu H, Shimizu S, Yamamoto M*, et al.* Expression of CD133 confers malignant potential by regulating metalloproteinases in human hepatocellular carcinoma. *J Hepatol* 2010, **52**(6)**:** 872-879.

3. Munz M, Baeuerle PA, Gires O. The emerging role of EpCAM in cancer and stem cell signaling. *Cancer Res* 2009, **69**(14)**:** 5627-5629.

4. Yang XR, Xu Y, Yu B, Zhou J, Li JC, Qiu SJ*, et al.* CD24 is a novel predictor for poor prognosis of hepatocellular carcinoma after surgery. *Clin Cancer Res* 2009, **15**(17)**:** 5518-5527.

5. Yang W, Wang C, Lin Y, Liu Q, Yu LX, Tang L*, et al.* OV6(+) tumor-initiating cells contribute to tumor progression and invasion in human hepatocellular carcinoma. *J Hepatol* 2012, **57**(3)**:** 613-620.

6. Xu J, Tan Y, Shao X, Zhang C, He Y, Wang J*, et al.* Evaluation of NCAM and c-Kit as hepatic progenitor cell markers for intrahepatic cholangiocarcinomas. *Pathol Res Pract* 2018, **214**(12)**:** 2011-2017.

7. Haraguchi N, Ishii H, Mimori K, Tanaka F, Ohkuma M, Kim HM*, et al.* CD13 is a therapeutic target in human liver cancer stem cells. *J Clin Invest* 2010, **120**(9)**:** 3326-3339.

8. Sun L, Zhang L, Chen J, Li C, Sun H, Wang J*, et al.* Activation of Tyrosine Metabolism in CD13+ Cancer Stem Cells Drives Relapse in Hepatocellular Carcinoma. *Cancer Res Treat* 2020, **52**(2)**:** 604-621.

9. Yang ZF, Ho DW, Ng MN, Lau CK, Yu WC, Ngai P*, et al.* Significance of CD90+ cancer stem cells in human liver cancer. *Cancer Cell* 2008, **13**(2)**:** 153-166.

10. Jia Q, Zhang X, Deng T, Gao J. Positive correlation of Oct4 and ABCG2 to chemotherapeutic resistance in CD90(+)CD133(+) liver cancer stem cells. *Cell Reprogram* 2013, **15**(2)**:** 143-150.

11. Fernando J, Malfettone A, Cepeda EB, Vilarrasa-Blasi R, Bertran E, Raimondi G*, et al.* A mesenchymal-like phenotype and expression of CD44 predict lack of apoptotic response to sorafenib in liver tumor cells. *Int J Cancer* 2015, **136**(4)**:** E161-172.

12. Dang H, Steinway SN, Ding W, Rountree CB. Induction of tumor initiation is dependent on CD44s in c-Met⁺ hepatocellular carcinoma. *BMC Cancer* 2015, **15:** 161.

13. Chiba T, Seki A, Aoki R, Ichikawa H, Negishi M, Miyagi S*, et al.* Bmi1 promotes hepatic stem cell expansion and tumorigenicity in both Ink4a/Arf-dependent and -independent manners in mice. *Hepatology* 2010, **52**(3)**:** 1111-1123.

14. Shan J, Shen J, Liu L, Xia F, Xu C, Duan G*, et al.* Nanog regulates self-renewal of cancer stem cells through the insulin-like growth factor pathway in human hepatocellular carcinoma. *Hepatology* 2012, **56**(3)**:** 1004-1014.

15. Xiang DM, Sun W, Zhou T, Zhang C, Cheng Z, Li SC*, et al.* Oncofetal HLF transactivates c-Jun to promote hepatocellular carcinoma development and sorafenib resistance. *Gut* 2019, **68**(10)**:** 1858-1871.

16. Ali N, Nguyen CB, Chandrakesan P, Wolf RF, Qu D, May R*, et al.* Doublecortin-like kinase 1 promotes hepatocyte clonogenicity and oncogenic programming via non-canonical β-catenin-dependent mechanism. *Sci Rep* 2020, **10**(1)**:** 10578.

17. Ma XL, Hu B, Tang WG, Xie SH, Ren N, Guo L*, et al.* CD73 sustained cancer-stem-cell traits by promoting SOX9 expression and stability in hepatocellular carcinoma. *J Hematol Oncol* 2020, **13**(1)**:** 11.

18. Bianco C, Rangel MC, Castro NP, Nagaoka T, Rollman K, Gonzales M*, et al.* Role of Cripto-1 in stem cell maintenance and malignant progression. *Am J Pathol* 2010, **177**(2)**:** 532-540.

19. Liu S, Li N, Yu X, Xiao X, Cheng K, Hu J*, et al.* Expression of intercellular adhesion molecule 1 by hepatocellular carcinoma stem cells and circulating tumor cells. *Gastroenterology* 2013, **144**(5)**:** 1031-1041 e1010.

20. Lo J, Lau EY, Ching RH, Cheng BY, Ma MK, Ng IO*, et al.* Nuclear factor kappa B-mediated CD47 up-regulation promotes sorafenib resistance and its blockade synergizes the effect of sorafenib in hepatocellular carcinoma in mice. *Hepatology* 2015, **62**(2)**:** 534-545.

21. Akbari S, Kunter I, Azbazdar Y, Ozhan G, Atabey N, Firtina Karagonlar Z*, et al.* LGR5/R-Spo1/Wnt3a axis promotes stemness and aggressive phenotype in hepatoblast-like hepatocellular carcinoma cell lines. *Cell Signal* 2021, **82:** 109972.

22. Wang Q, Liang N, Yang T, Li Y, Li J, Huang Q*, et al.* DNMT1-mediated methylation of BEX1 regulates stemness and tumorigenicity in liver cancer. *J Hepatol* 2021.

23. Tamai K, Nakamura-Shima M, Shibuya-Takahashi R, Kanno SI, Yasui A, Mochizuki M*, et al.* BEX2 suppresses mitochondrial activity and is required for dormant cancer stem cell maintenance in intrahepatic cholangiocarcinoma. *Sci Rep* 2020, **10**(1)**:** 21592.

24. Liu L, Dai Y, Chen J, Zeng T, Li Y, Chen L*, et al.* Maelstrom promotes hepatocellular carcinoma metastasis by inducing epithelial-mesenchymal transition by way of Akt/GSK-3β/Snail signaling. *Hepatology (Baltimore, Md)* 2014, **59**(2)**:** 531-543.

25. Huang L, Cai J, Guo H, Gu J, Tong Y, Qiu B*, et al.* ID3 Promotes Stem Cell Features and Predicts Chemotherapeutic Response of Intrahepatic Cholangiocarcinoma. *Hepatology* 2019, **69**(5)**:** 1995-2012.

26. Xu X, Liu RF, Zhang X, Huang LY, Chen F, Fei QL*, et al.* DLK1 as a potential target against cancer stem/progenitor cells of hepatocellular carcinoma. *Mol Cancer Ther* 2012, **11**(3)**:** 629-638.

27. Yu F, Hao X, Zhao H, Ge C, Yao M, Yang S*, et al.* Delta-like 1 contributes to cell growth by increasing the interferon-inducible protein 16 expression in hepatocellular carcinoma. *Liver Int* 2010, **30**(5)**:** 703-714.

28. Zhao W, Wang L, Han H, Jin K, Lin N, Guo T*, et al.* 1B50-1, a mAb raised against recurrent tumor cells, targets liver tumor-initiating cells by binding to the calcium channel α2δ1 subunit. *Cancer Cell* 2013, **23**(4)**:** 541-556.

29. Wei X, Zhao L, Ren R, Ji F, Xue S, Zhang J*, et al.* MiR-125b Loss Activated HIF1alpha/pAKT Loop, Leading to Transarterial Chemoembolization Resistance in Hepatocellular Carcinoma. *Hepatology* 2021, **73**(4)**:** 1381-1398.

30. Gu Y, Wei X, Sun Y, Gao H, Zheng X, Wong LL*, et al.* miR-192-5p Silencing by Genetic Aberrations Is a Key Event in Hepatocellular Carcinomas with Cancer Stem Cell Features. *Cancer Res* 2019, **79**(5)**:** 941-953.

31. Li J, Zhong X, Wang X, Xu F, Yang J, Lu J*, et al.* miR-93 regulates liver tumor initiating cells expansion and predicts chemotherapeutic response of patients. *Arch Biochem Biophys* 2021, **703:** 108871.

32. Li L, Liu Y, Guo Y, Liu B, Zhao Y, Li P*, et al.* Regulatory MiR-148a-ACVR1/BMP circuit defines a cancer stem cell-like aggressive subtype of hepatocellular carcinoma. *Hepatology* 2015, **61**(2)**:** 574-584.

33. Chai S, Ng KY, Tong M, Lau EY, Lee TK, Chan KW*, et al.* Octamer 4/microRNA-1246 signaling axis drives Wnt/beta-catenin activation in liver cancer stem cells. *Hepatology* 2016, **64**(6)**:** 2062-2076.

34. Wu J, Zhu P, Lu T, Du Y, Wang Y, He L*, et al.* The long non-coding RNA LncHDAC2 drives the self-renewal of liver cancer stem cells via activation of Hedgehog signaling. *J Hepatol* 2019, **70**(5)**:** 918-929.

35. Wang X, Sun W, Shen W, Xia M, Chen C, Xiang D*, et al.* Long non-coding RNA DILC regulates liver cancer stem cells via IL-6/STAT3 axis. *J Hepatol* 2016, **64**(6)**:** 1283-1294.

36. Wang Y, He L, Du Y, Zhu P, Huang G, Luo J*, et al.* The long noncoding RNA lncTCF7 promotes self-renewal of human liver cancer stem cells through activation of Wnt signaling. *Cell Stem Cell* 2015, **16**(4)**:** 413-425.

37. Shao M, Yang Q, Zhu W, Jin H, Wang J, Song J*, et al.* LncHOXA10 drives liver TICs self-renewal and tumorigenesis via HOXA10 transcription activation. *Mol Cancer* 2018, **17**(1)**:** 173.

38. Zhu P, Wang Y, Wu J, Huang G, Liu B, Ye B*, et al.* LncBRM initiates YAP1 signalling activation to drive self-renewal of liver cancer stem cells. *Nat Commun* 2016, **7:** 13608.

39. Zhu YJ, Zheng B, Luo GJ, Ma XK, Lu XY, Lin XM*, et al.* Circular RNAs negatively regulate cancer stem cells by physically binding FMRP against CCAR1 complex in hepatocellular carcinoma. *Theranostics* 2019, **9**(12)**:** 3526-3540.

40. Chen L, Shi J, Wu Y, Qiu R, Zeng L, Lou L*, et al.* CircRNA CDR1as promotes hepatoblastoma proliferation and stemness by acting as a miR-7-5p sponge to upregulate KLF4 expression. *Aging (Albany NY)* 2020, **12**(19)**:** 19233-19253.

41. Wei Y, Chen X, Liang C, Ling Y, Yang X, Ye X*, et al.* A Noncoding Regulatory RNAs Network Driven by Circ-CDYL Acts Specifically in the Early Stages Hepatocellular Carcinoma. *Hepatology* 2020, **71**(1)**:** 130-147.

42. Sun L, Wang Y, Cen J, Ma X, Cui L, Qiu Z*, et al.* Modelling liver cancer initiation with organoids derived from directly reprogrammed human hepatocytes. *Nat Cell Biol* 2019, **21**(8)**:** 1015-1026.

43. Bai HY, Liao YJ, Cai MY, Ma NF, Zhang Q, Chen JW*, et al.* Eukaryotic Initiation Factor 5A2 Contributes to the Maintenance of CD133(+) Hepatocellular Carcinoma Cells via the c-Myc/microRNA-29b Axis. *Stem Cells* 2018, **36**(2)**:** 180-191.

44. Wang J, Dong M, Xu Z, Song X, Zhang S, Qiao Y*, et al.* Notch2 controls hepatocyte-derived cholangiocarcinoma formation in mice. *Oncogene* 2018, **37**(24)**:** 3229-3242.

45. Wang R, Li Y, Tsung A, Huang H, Du Q, Yang M*, et al.* iNOS promotes CD24(+)CD133(+) liver cancer stem cell phenotype through a TACE/ADAM17-dependent Notch signaling pathway. *Proc Natl Acad Sci U S A* 2018, **115**(43)**:** E10127-E10136.

46. Raggi C, Invernizzi P, Andersen JB. Impact of microenvironment and stem-like plasticity in cholangiocarcinoma: molecular networks and biological concepts. *J Hepatol* 2015, **62**(1)**:** 198-207.

47. Leung HW, Leung CON, Lau EY, Chung KPS, Mok EH, Lei MML*, et al.* EPHB2 Activates beta-Catenin to Enhance Cancer Stem Cell Properties and Drive Sorafenib Resistance in Hepatocellular Carcinoma. *Cancer Res* 2021, **81**(12)**:** 3229-3240.

48. Chang WH, Lai AG. Aberrations in Notch-Hedgehog signalling reveal cancer stem cells harbouring conserved oncogenic properties associated with hypoxia and immunoevasion. *Br J Cancer* 2019, **121**(8)**:** 666-678.

49. Tang Y, Kitisin K, Jogunoori W, Li C, Deng CX, Mueller SC*, et al.* Progenitor/stem cells give rise to liver cancer due to aberrant TGF-beta and IL-6 signaling. *Proc Natl Acad Sci U S A* 2008, **105**(7)**:** 2445-2450.

50. Song M, He J, Pan QZ, Yang J, Zhao J, Zhang YJ*, et al.* Cancer-Associated Fibroblast-Mediated Cellular Crosstalk Supports Hepatocellular Carcinoma Progression. *Hepatology* 2021, **73**(5)**:** 1717-1735.
