# Supplementary material for: Mechanism of cancer stemness maintenance in human liver cancer
Source: Cell Death Dis. 2022 Apr 21;13(4):394. doi: 10.1038/s41419-022-04848-z (PMC9023565; doi:10.1038/s41419-022-04848-z)
Supplement: Supplementary file 2 — Language Editing Certificate [file 41419_2022_4848_MOESM2_ESM.pdf]

ELSEVIER

Certificate of Elsevier Language Editing Services

The following article was edited by Elsevier Language Editing Services:

"Mechanism of cancer stemness maintenance in human liver cancer"

Authored by:

Xianli He

Date: 18-Feb-2022

Serial number: LE-232943-432D229C322C
